# An EEG/EMG/EOG-Based Multimodal Human-Machine Interface to Real-Time Control of a Soft Robot Hand

**DOI:** 10.3389/fnbot.2019.00007

**Published:** 2019-03-29

**Authors:** Jinhua Zhang, Baozeng Wang, Cheng Zhang, Yanqing Xiao, Michael Yu Wang

**Affiliations:** ^1^Key Laboratory of Education Ministry for Modern Design and Rotor-Bearing System, School of Mechanical Engineering, Xi'an Jiaotong University, Xi'an, China; ^2^School of Biological Science and Medical Engineering, Beihang University, Beijing, China; ^3^Departments of Mechanical and Aerospace Engineering and Electronic and Computer Engineering, HKUST Robotics Institute, Hong Kong University of Science and Technology, Kowloon, Hong Kong

**Keywords:** electroencephalogram (EEG), electromyogram (EMG), electrooculogram (EOG), multimodal human-machine interface (mHMI), soft robot hand

## Abstract

Brain-computer interface (BCI) technology shows potential for application to motor rehabilitation therapies that use neural plasticity to restore motor function and improve quality of life of stroke survivors. However, it is often difficult for BCI systems to provide the variety of control commands necessary for multi-task real-time control of soft robot naturally. In this study, a novel multimodal human-machine interface system (mHMI) is developed using combinations of electrooculography (EOG), electroencephalography (EEG), and electromyogram (EMG) to generate numerous control instructions. Moreover, we also explore subject acceptance of an affordable wearable soft robot to move basic hand actions during robot-assisted movement. Six healthy subjects separately perform left and right hand motor imagery, looking-left and looking-right eye movements, and different hand gestures in different modes to control a soft robot in a variety of actions. The results indicate that the number of mHMI control instructions is significantly greater than achievable with any individual mode. Furthermore, the mHMI can achieve an average classification accuracy of 93.83% with the average information transfer rate of 47.41 bits/min, which is entirely equivalent to a control speed of 17 actions per minute. The study is expected to construct a more user-friendly mHMI for real-time control of soft robot to help healthy or disabled persons perform basic hand movements in friendly and convenient way.

## Introduction

Stroke is ranked as the third most common cause of disability worldwide and seriously affects the quality of life of survivors in terms of their daily functioning (Lim et al., 2012). Up to 80% of stroke survivors are left with a residual deficit in movement function of the arm and hand (Hung et al., 2016). It has been found that rehabilitation is most effective if instituted early on the first 6 months post-stroke, when the mechanisms of brain plasticity are more active and facilitate relearning and recovery of hand function (Robertson and Murre, 1999). Although a rehabilitation program involving repetitive movements of the activities of daily living can allow stroke survivors to partially recover lost motor function, it is difficult for many patients to move their affected upper limbs in the manner required by such physical training routines (Ang and Guan, 2013). Many clinical studies have indicated that active, repetitive, and intensive rehabilitation training may have significant benefits for the recovery of impaired motor functions (Fisher and Sullivan, 2001; Schaechter, 2004). In the traditional therapeutic approach, physical therapists teach stroke survivors how to guide their movements with the aim of regaining basic physical skills. However, this approach is highly labor-intensive, inefficient, and requires a good deal of physical effort on the part of patients, who may sometimes refuse to actively cooperate with the regime. In addition, patients may need to be hospitalized for some of their rehabilitation. Another problem is that many physical therapists may not have received the necessary training to prepare them to administer such stroke rehabilitation programs (Curtis and Martin, 1993). Furthermore, the process of rehabilitation training is inadequate in that it does not deal with Brain–computer interfaces (BCI). The above factors have severely restricted the clinical effectiveness of rehabilitation training.

Recently, robot-assisted physical therapy has been proposed to enhance neurological rehabilitation in traditional post-stroke therapy. Specifically, not only can robotic devices be timed to provide rehabilitation training for long periods, delivering a suitable force for patients in a consistent, precise manner, without fatigue, but they can also be programmed to switch between different therapeutic modes depending on the state of rehabilitation of the patient. They are also able to monitor and record patients' performance during rehabilitation training (Takahashi et al., 2008). Many clinical studies have indicated that robot assistance can significantly enhance the performance of physical therapy involving intensive repetitive hand movements aimed at improving limb function (Fasoli et al., 2003). The human hand is a delicate and intricate structure made up of a total of 27 individual bones, and its joints allow a wide range of precise movements with around 21 degrees of freedom (DOF) and subject to a complex distribution of forces. Although conventional rigid robots are able to deliver linear and rotational motion to the limbs of stroke patients with high forces and torques, they still have some shortcomings. Typically, they are heavy, noisy, and expensive; they suffer from limited adaptability; they are potentially unsafe; and they require care and time for proper alignment with human joints (Polygerinos et al., 2015). Soft hand robots have a number of advantages over conventional robotic devices. They have a continuously deformable structure that fits snugly with the fingers, allowing accurate performance of exercises. They also allow plastic bending with a high degree of curvature and a high level of security, and there are positive interactions between limb and robot. As a result, the painful muscle cramps or spasms and secondary injury that can occur with robotic systems are effectively avoided (Rus and Tolley, 2015). With the use of soft hand robots, stroke patients are able to participate actively in rehabilitation exercises that involve bending motion of the fingers to meet basic requirements of everyday life, such as drinking and eating. However, the appropriate control strategy to make a soft robot comply with a subject's intended motion is still an open problem.

BCI have the potential to provide an assistive technology that converts brain activity into commands communicating with a user's intent to control robot-assisted system that promote the neural plasticity required for recovery of function after stroke (Wolpaw et al., 2002).

The method combining hybrid BCI and robot-assisted therapy is more effective to recover from stroke help activate brain plasticity than single traditional rehabilitation therapy (Dipietro et al., 2005; Ang et al., 2015). Furthermore, the control commands can be based on features extracted from biological signals, such as electroencephalography (EEG), electrooculography (EOG), and electromyography (EMG). For example, using advanced methods for detection, processing, and classification of EMG signals from muscle movements, it has proved possible to drive a prosthetic hand with fast response and high precision (Gray et al., 2012). However, stroke can lead to muscle weakness to such an extent that muscles cannot produce adequate forces for effective classification of the resulting EMG signals, thus limiting the clinical application of this approach (Lum et al., 2012). An alternative approach is to monitor EEG activity as a patient imagines an intended movement, thereby exploiting neural informations as input control for a robotic prosthesis. Unfortunately, EEG signals do not have sufficient spatial resolution for them to be used to control individual finger movements. Besides, EEG signals are attenuated during transmission, which hampers post-classification processing of these signals to control fine movements (Xiao and Ding, 2013). EOG signals have good stability and larger potentials than EEG. EOG can be applied to BCI at quite low cost and provides good accuracy, so this is another potentially useful method for controlling robotic prostheses. However, the application of this approach over sustained periods is limited by the fact that users' eyes tend to become dry, fatigued, or even painful (Singh and Singh, 2012). Each of these traditional single-mode BCI systems based on EEG, EOG, or EMG has its own disadvantages hindering further development. In order to make use of the respective advantages of the different types of BCI, it is possible to combine different modes in an approach called multi-modal HMI (mHMI) (Allison et al., 2010). However, whether single- or double-mode, these methods of active control still possess a number of shortcomings, such as a limited number of possible commands, poor real-time capability, and failure to meet the requirements of the basic actions required in rehabilitation training.

The aim of mMHI combining two or more user modes such as eye movements, hand gestures, and motor imagery in a coordinated approach is to increase the number of instructions and enhance classification accuracy, reduce errors, and overcome the specific disadvantages of each individual mode of BCI (Amiri et al., 2013; Zhang et al., 2016). For example, Edlinger et al. introduced a system employing real-time analysis of EEG, EMG, EOG, and motion sensors to implement three different types of navigation optimally suited to a user's needs for a specific control task. However, this system required subjects to perform predefined tasks in chronological order (Edlinger et al., 2013). Nam et al. presented a novel HMI that allowed a user to control a humanoid robot by selecting items from a predefined menu through eye and tongue movements and tooth clenching detected by GKP (glossokinetic potential), EOG, and EMG signals, respectively (Nam et al., 2014). This suggests the possibility of an mHMI approach that integrates two or more brain/nonbrain signal acquisition modalities from areas other than the damaged hemisphere. There have also been some proposals for simple switches and motion sensors in hybrid EEG–EOG based BCI (Lalitharatne et al., 2013). However, to the best of our knowledge, there is still no relatively mature, practical method of mHMI that integrates EEG, EOG, and EMG for on-line control of robot-assisted system for stroke rehabilitation training.

On the basis of recent research on mMHI, this study introduces a three-mode interface that should allow normal subjects to control soft hand robot performing intense repetitive hand movements. The system can recognize motor imagery, hand gestures, and eye movements. EEG based pattern is used to detect the intention of left or right hand movement. EMG-based pattern is used to identify hand gestures to facilitate control of the robot. EOG-based pattern is used not only to recognize eye movements such as looking left and right, but also, by double blinking of the eyes, to select different actions that best suit the subject's needs within a selected category. Given high performance in mHMI, this is a critical step in the development of an effective, mature, practical training system for motor functional recovery.

## Methods and Materials

### Participants

Six healthy subjects (four men and two women, aged 23–26 years old, and all right handed) were recruited to participate in the study at regular times during their work period. All but one of the subjects had prior experience with mHMI or similar experiments. The subjects were all able to control the mHMI system with their intentions and use their hands for any activity of daily living. More than anything, all signed an informed consent forms after having been notified for the experimental procedure. And the study was approved by the Ethics Committee of Xi'an Jiaotong University, China, and they were managed according to the ethical standards of the latest Declaration of Helsinki.

### Experiment Apparatus and Setup

**Figure 1:**
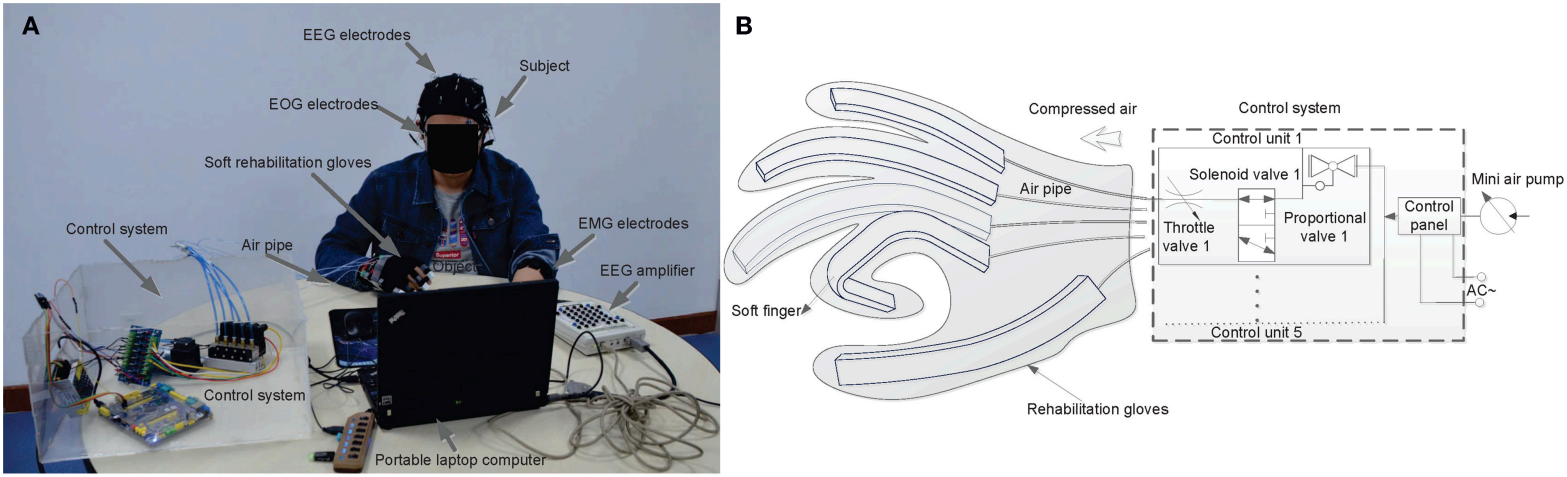
Display of the experimental condition and control system to demonstrate the using principles of mHMI in hand testing process, **(A)** is the prototype model of mHMI and experimental condition, **(B)** is the schematic of control system and soft robot hand.

The proposed prototype mHMI combines EEG, EOG, and EMG modes into a fully integrated system to allow handicapped people to control their peripheral mobility. Each subject was required to sit comfortably watching the 14-inch screen of a portable laptop computer (Windows 7, Intel (R) Core (TM) i7 CPU, 2.10 GHz, 2.70 GHz, 3.19 GB RAM, and 32 bits operational system), with a Myo Armband (Thalmic Labs Inc., USA) on one forearm to track arm movements while the other arm pulled on the soft robot as shown in Figure 1. The subject was asked to rest both arms comfortably on the desk, which was in its own room to reduce noise and distractions.

The experiment was carried out to control soft robot as shown in Figure 1A, EEG and EOG signals were recorded using a Neuroscan NuAmps Express system (Compumedics Ltd., VIC, Australia). An elastic cap with 40 mounted Ag/AgCl electrodes positioned according to the 10–20 international system was used to detect EEG activities and EOG movements, with all the electrode impedances being kept below 5 k. Both EEG and EOG were sampled at 500 Hz with the electrode (A2) on the right mastoid acting as reference and the electrode (GND) on the forehead as ground. EMG were used to track arm movements which were obtained from forearm muscle activities through the Myo Armband, with eight EMG sensors, a gyroscope, an accelerometer, and a magnetometer measuring muscle tension traveling across the widest part of the user's healthy arm. The EMG electrode impedance was maintained below 20 k. The Myo was capable of collecting EMG at a sampling rate of 200 Hz, and employed wireless data communication (Bluetooth) with its own dongle. The EEG, EOG, and EMG signals were simultaneously recorded, and a notch filter was used to remove 50 Hz interference. The soft robot was a custom-built device developed and produced by our team for neuro-motor rehabilitation of normal hands, as shown in Figure 1B. The details of its design and the associated experimental setup can be found elsewhere (Zhang et al., 2015). The robot comprised a lightweight comfortable glove, electric actuators, a control panel, and a mini air pump, and was able to safely execute all combinations of joint flexion-extension. Each actuator was linked to a PVC pipe connected to an air pump to apply air pressure through the control unit. The control unit included proportional valves, a throttle valve, solenoid valves and other components. Flexible finger movements were made possible by five flexion actuators worn on the hand and connected through an electronic board.

### Experimental Procedure

**Figure 2:**
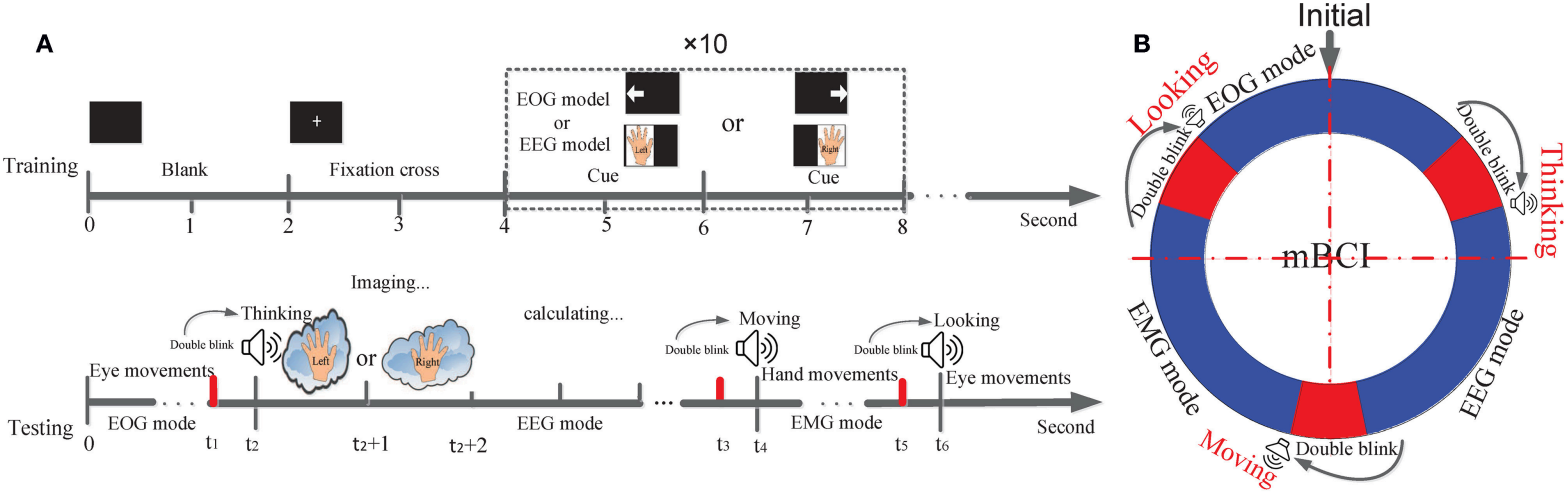
Trial structure for training and testing paradigm and process of mode-alternating. **(A)** Trial procedure of a training and testing phase according to the cue presented in the screen or emitted from the computer. The time ranges of EOG, EEG and EMG mode are from 0 to t1, from t2 to t3, and from t4 to t5, respectively. The time ranges for mode-alternating separately are from t1 to t2, from t3 to t4, and from t5 to t6. **(B)** The mode-alternating circular ring describes the mode-alternating process of the three modes according to subject's intention from double blink.

The experimental approach was similar to that adopted in similar mHMI studies (Ma et al., 2015; Minati et al., 2016) and illustrated in Figure 2. It involved a training phase and a testing phase, as depicted in Figure 2A. Subjects were asked to spend <2 min carrying out the training to set the parameters for the EOG and EEG modes simultaneously, since they were known to be familiar with the experiment. The screen was black for the first 2 s, then a cross appeared in the center of the screen until 4 s, after which a cue picture appeared in a dashed border for 2 s. In the EOG mode, the appearance of a left or right arrow instructed the subject to track the arrow with their eyes (looking left and right) and with their eyes blinking naturally. In the EEG mode, imagined left or right hand movement appeared in turn on the screen as a cue demonstrating the motor imagery of the corresponding hand movement for 2 s. Both the EOG and EEG modes involved 10 trials, including 5 left arrows and 5 right arrows, or 5 times left hand motor imagery (MI), and 5 times right hand MI. All the subjects were asked to track the left or right arrows or imagine either left hand MI or right hand MI, depending on a sequential visual cue stimulus. Thus, each trial of the EOG mode or EEG mode lasted for 44 s.

Moreover, in EMG mode, subjects were required to create a custom profile with the proper guidance of MYO Windows software development kit (MYO SDK) 1.0.1 for Windows 7 that is free to download from the website (Ganiev et al., 2016; Labs, 2018b).

After completion of a training phase, the mHMI enters a testing phase. Figure 2B is a ring chart illustrating the alternation of the three modes (EOG, EEG, and EMG). This mode-alternating cycle makes it possible to control the entire system with high efficiency according to the user's intentions. In the initial condition of the mHMI system, the EOG mode is adopted as the default. Because this mode works in an asynchronous fashion, the system is always able to actively detect eye movements, and the user can repeatedly change and send EOG instructions to the soft robot at any time. If the system is idle, double blinks, which have the highest priority, can switch it to the next mode no matter what its current mode is. The EEG mode, in contrast, operates synchronously. When a double blink is detected using EOG, the system enters the EEG mode automatically and the computer emits a “Thinking” sound (not too loud). The subject then begins to imagine the execution of left or right hand movement (Jongsma et al., 2013), and the output data are stored in a standard text file. The system can then automatically issue the appropriate commands to control the soft robot using our customized software. When the system is idle, double blinks can again switch mode, in this case to the EMG mode, accompanied by a “Moving” sound. The robot is then controlled by commands generated by the software from EMG, which works in an asynchronous fashion. Again, at any time, double blinks can make the system reenter the EOG mode, accompanied by a “Looking” sound, thus completing the alternation of the three modes.

After setting the mHMI parameters, an experiment involving real-time control of the soft robot is performed to verify the system performance. Subjects are again required to rest their arms comfortably on the desk as in training, but this time listening for cue sounds without looking at the computer screen. They are able to control the soft robot using only their minds in accordance with whichever one of a set of specific soft hand actions that they would like to execute. There is a customized correspondence between five basic hand gestures and five hand tasks. Those hand gestures that are more easily performed correspond to the hand actions that are most frequently used and are most important. The optimum relationship between hand gestures and actions and these hand actions are all listed in Table 1. In each session of the experiment, a subject performs all the actions for each mode. When the mHMI and user are idle, the subject is able to switch to next mode. All the subjects implemented a single session with 50 runs, resting for 3~5 min between each run. The whole process is captured on videotape to record the time and the number of correct and wrong actions.

**Table 1:** A series of types of hand movement, task and hand action descriptions are presented in different modes for the proposed mHMI.

**Modes**	**Types of hand movement**	**Task**	**Hand action**
EOG	Non grip	Look left	Push with bend five fingers
		Look right	Pull with pinch five fingers
		Double blink	Modal shift
EEG	Power grip	Left hand MI	Three finger grip
		Right hand MI	Fist
EMG	precision grip	Rest	Idle
		Fist	Ball pinch
		Wave in	Tip pinch
		Wave out	Multiple-tip pinch
		Fingers spread	Finger loosen up
		Double tap	Cylindroids grip

### Detection of Movement Intention Using mHMI

**Figure 3:**
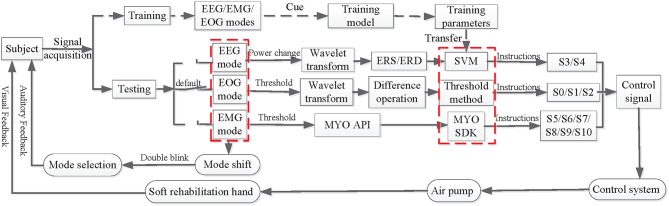
The flow charts of movement intention detection and controlling soft robot using the mHMI.

In a testing phase, many control scenarios demand real-time and multitasking control commands detected from the user's intention. Figure 3 shows how the mHMI system is based on a combination of event-related desynchronization (ERD) and synchronization (ERS), eye movements and hand gestures, making full use of the advantages of each mode and helping to overcome the disadvantages. The most significant aspect of the mHMI is its versatility and flexibility, as represented by the various hand actions. Before detecting user's intention, the training model of EOG and EEG are built by the training data collecting 10 times trials per mode to calculate thresholds and train classifier parameters, respectively. Then the MYO must be warm up and performed a special calibration hand gestures according to the requirements of MYO API every time in case of re-positioning the MYO on user's arm. For the calibration and successful synchronization, the API is able to accurately calculate a user's custom profile whose related parameters are saved in a computer.

Once the mHMI enters a testing stage, in EOG mode, EOG are firstly filtered using a zero-phase FIR lowpass filter (hamming window) with a lower cutoff frequency 0.05 Hz and a higher cutoff frequency 45 Hz (Shao et al., 2017). Next, the results are obtained by filtered EOG based on wavelet transform using the 4th order of Daubechies Wavelet with 10 decomposition levels. So EOG model is established using the dual threshold method to identify eye movement of look left, look left and double blink, and transform them into corresponding commands which separately are push with bend five fingers (S1), pull with pinch five fingers (S2), and modal shift (S0). In EEG mode, the DC component and baseline drift with respect to a preferred common average reference are first removed from the EEG signals and they are filtered by a zero-phase FIR lowpass filter with cut off frequency 0.05~45 Hz. Next, the grand averaged ERD/ERS in the EEG are estimated as the power decrease (ERD) or power increase (ERS) compared with first 2 s reference interval of the resting state (Tang et al., 2016; Cho et al., 2017). The mean values of the quadratic sums of the signals separately obtained by left or right hand MI are calculated. The power changes in each channel are extracted through Daubechies wavelet at 5 levels using the db4 mother wavelet for the mean value (Chen et al., 2017). The difference between the mean values from the EEGs recorded respectively on the central region positioned over the left (C3) and right (C4) primary sensorimotor cortex is obtained (Babiloni et al., 2008), and the ERD and ERS are calculated using difference normalization. An optimal hyper-plane is then constructed as the decision surface for two-class feature classification, following which the appropriate kernel function RBF is select, and the parameters of the decision surface are determined according to the principles of Support Vector Machines (SVM) using MATLAB functions “svmtrain.m” under Matlab 2010a (MathWorks, Inc.) (Chang and Lin, 2011; Lawhern et al., 2012). Final, the processed EEG data are input to the trained model of the SVM classifier, and the classification result is then obtained from the test model on the testing set. The results are easily translated into three fingers grip (S3) and fist (S4) command to control the soft robot. In EMG mode, if most dominant arm muscles are alive, a subject can wear MYO on his/her arm and practice the five basic hand gestures (Ganiev et al., 2016). The details of all operation also can be found at MYO support in the website (Labs, 2018a). The application programming interface of Myo Connect is used to real-time obtain gestural data from EMG activities of forearm, and EMG are input into the packaged pattern recognition algorithm to classify hand gestures which are successfully transformed to corresponding control instructions (such as S5, S6, S7, S8, S9, and S10).

### On-Line Control Soft Robot Hand With the mHMI

**Figure 4:**
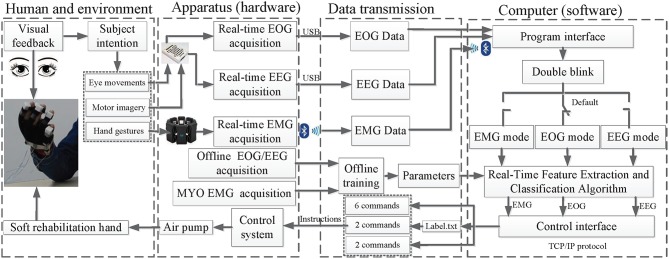
The main structure and work flow of the mHMI.

The prototype mHMI has the advantages of friendly human-machine interaction and efficient real-time control of the soft robot (Martišius and Damaševičius, 2016). The system is implemented based on our customized C++ application, which has been developed to allow on-line recording of EOG, EEG, and EMG while ensuring that all data remain synchronized; the details of the similar synchronization method are described in the references (Luu et al., 2017). As shown in Figure 4, there are four aspects to the achievement of this goal.

The first aspect is human and environmental. The subject can select different mental/action tasks in accordance with the external environment, such as eating or drinking, to control the soft robot, after which the outputs of the control are fed back visually in real time to the user. This enhances system's interaction ability, and might be effective for hand recovery in patients with hand muscle weakness. The second aspect is the provision of hardware support for an on-line/off-line data acquisition and control system. The training model needs to collect EOG and EEG using the same NuAmps system as for training data, following which the calculated correlation parameters are transmitted to the testing model in an off-line training phase. In addition, a special calibration of hand gestures should be performed to generate a user's custom profile. The testing model then automatically calls the related parameters with the Myo API. In the on-line testing phase, live EOG and EEG are collected using the NeuroScan SDK, while live EMG data are collected using the Myo. The control system real-time receives intentions to control air pump through a communication line. The third aspect is the communication link between hardware and software, from which information can be exchanged between software, computer hardware, and soft robot. The EEG, EOG, EMG and the offline training parameter are entered into the computers by the program interface, with instructions being available directly from the control interface. The instructions for the EEG mode are written in a file named Label.txt, which is generated by calling a training program written in MATLAB. Each time the program is re-run, the previous Label.txt file is overwritten.

The fourth aspect is the software integration platform which employs multi-threading and multi-processing techniques for real-time data acquisition, pre-processing, feature extraction, and classification, with each subsystem for EEG, EOG, and EMG being contained in an individual thread (Bulea et al., 2013). The EOG mode is the default thread, but the program interface can allow switching between threads through double blinking. Last, but most importantly, the soft robot is controlled using the C language on a single-board computer and the C++ implemented in Visual Studio 2010 for hardware interfacing. Communication between commands generated by different modes and the control system is operated as a TCP/IP server, from which the program interface can transfer data reliably and can accept commands from the dedicated threads to allow real-time on-line implementation of the mHMI (Minati et al., 2016).

### Evaluation Criteria of the mHMI

To complete the evaluation of the mHMI, the performance is estimated in terms of classification accuracy (ACC), control speed, and average time in EOG, EEG, EMG mode and multimodal. ACC is defined as the percentage of successful actions. Control speed stands for the number of correct actions in 1 min. Average time presents mean time of whole task in 1 min. These parameters have been calculated previously as follows (López et al., 2017).

(1) $$\begin{array}{rcl} ACC & = & \frac{Correct\;action\;output}{Total\;number\;of\;sections}\times 100\% \end{array}$$

(2) $$\begin{array}{rcl} Speed & = & \frac{Number\;of\;correct\;actions}{Process\;time} \end{array}$$

In addition, to further assess overall classification performance, the *ACC, control speed* and the number of movement intention are combined by the information transfer ratio (*ITR*) which is used to calculate for all the movement intention of different modes (Djemal et al., 2016).

(3) $$\begin{array}{r} ITR=\\ S\times [log_{2}N+Acc\times log_{2}Acc+\left(1-Acc\right)\times log_{2}\left(\frac{1-Acc}{N-1}\right] \end{array}$$

Where *S* is control speed. *Acc* is classification accuracy of each mode. *N* is the number of possible movement intention.

## Results and Discussion

### Analysis of Movement Intention

**Figure 5:**
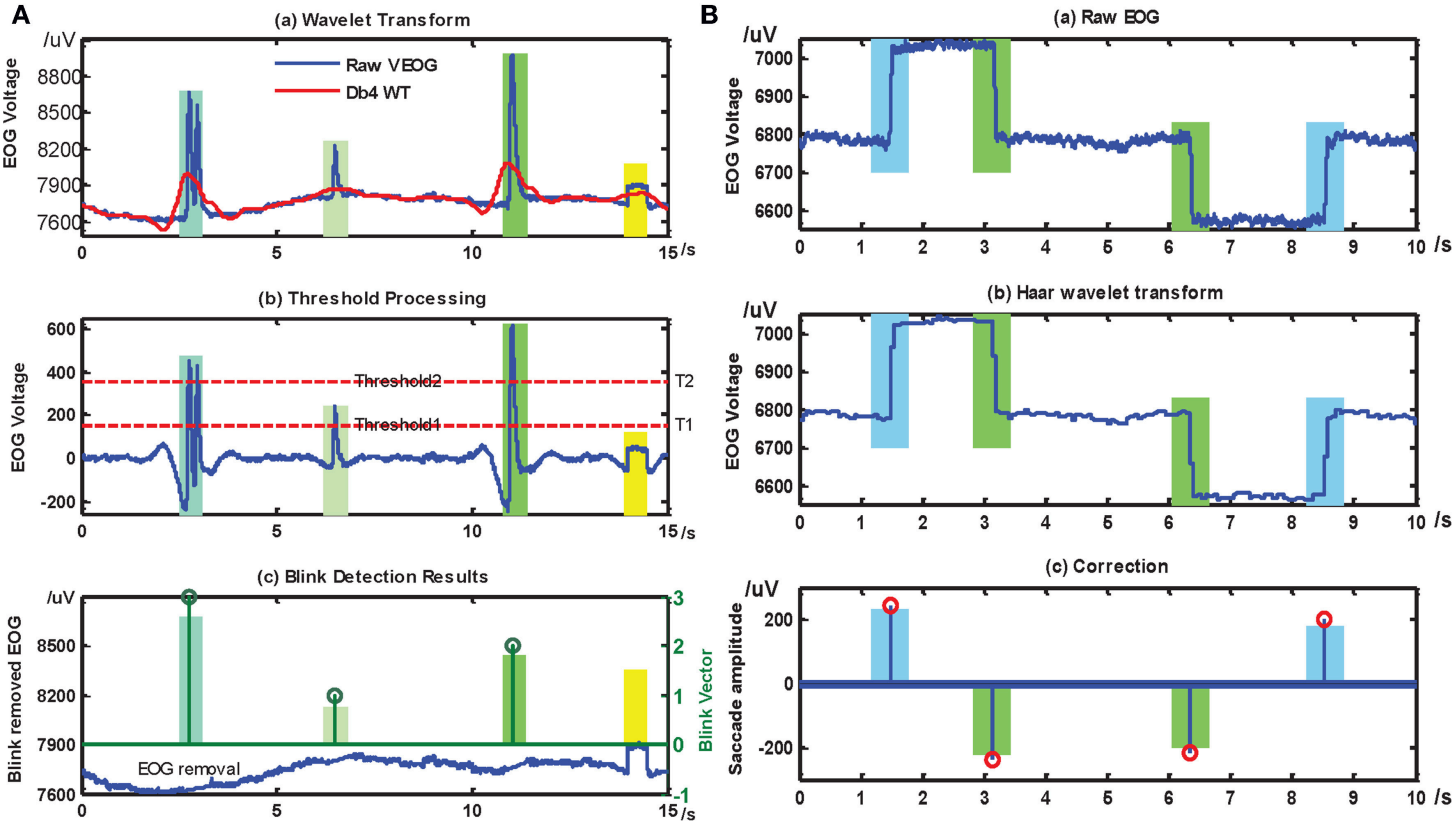
The recognition results of blinks and saccades detection. After treatment of EOG, the same section marked in same color with bar graph. **(A)** is the processing results of blink base on EOG, the threshold values 1, 2, and 3 of blink vector separately stand for an involuntary blink, a voluntary single blink and a voluntary double blink, **(B)** is the processing results of saccade, if the saccade amplitude is a positive number, it represents left saccade (blue), otherwise it means right saccade (green).

One of the advantages of the mHMI is to combine EOG, EEG, and EMG modes to detect movement intention, and the significant feature of movement intention are analyze and compare in each mode. In EOG mode, as shown in Figure 5A, an eye movement detection method is applied according to the different distributed voltage ranges. The first subgraph describes the differences from a comparison between the raw EOG and the signal after wavelet de-noising. In the second subgraph, if the signal is larger than the first threshold 180 μV, then the blink is taken to be significant; otherwise it is ignored. Further, if the signal is larger than the second threshold 385 μV, the blink is considered to be voluntary blink; otherwise, it is regarded as an involuntary blink. The final subgraph represents a blink removed EOG in the vertical direction and a separate voluntary blink. If the blink vector reaches or exceeds a threshold of 3, this is taken to indicate a voluntary double blink. Figure 5B depicts the process of saccade detection. The fist subgraph shows the raw EOG obtaining 4 saccade singles. These are then reconstructed based on wavelet transform in second one. A single-valued pulse signal can then be obtained from the point-wise difference of the signals, although some noisy random signals are decomposed into small stair-step signals, so a threshold 30 μV should be set to correctly identify pulse signals. If a threshold is met or exceeded, the pulse signal is either regarded as a saccade signal or is neglected. The corrected saccade signal is obtained as shown in the third subgraph: if the pulse signal is positive, it represents looking right; otherwise it represents looking left. After correct identification, the looking-left and looking-right signals can be transformed into commands for the robot hand to push with bend five fingers and to pull with pinch five fingers, and the double blink signal can be transformed into a command for the system to shift mode.

**Figure 6:**
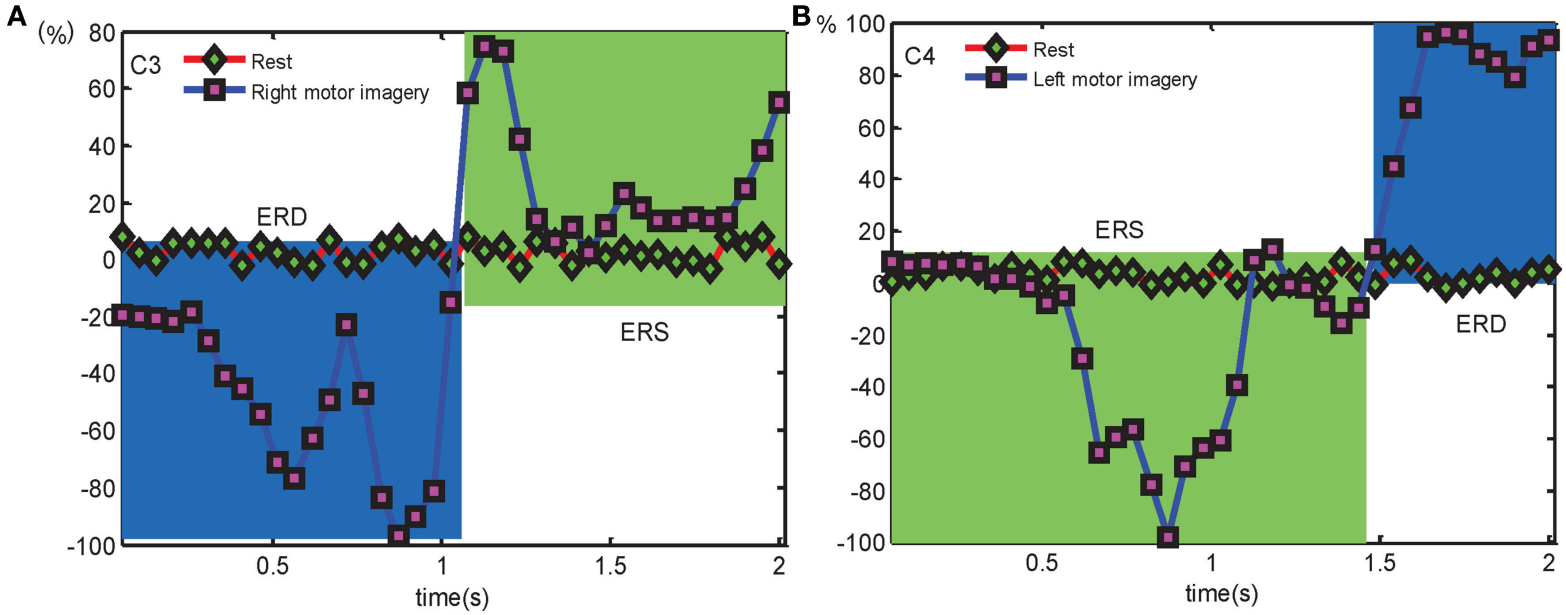
The grand average ERD/ERS over C3 and C4 electrodes separately compares to the corresponding rest condition for detecting left- or right hand MI, **(A)** is the ERD/ERS over C3 electrode in right hand MI condition, **(B)** is the ERD/ERS over C4 electrode in left hand MI condition.

In EEG mode, the physiological phenomena reflecting sensorimotor brain activity through ERD/ERS components are extracted from the EEG signal to identify different tasks. The grand average ERD/ERS of C3 and C4 in the frequency range of 8 ~ 12 Hz is calculated as compared to the reference period 2 s before the cue occurs across 10 trials for each subject. The mean ERD/ERS of left or right hand MI for different condition are visible in Figure 6. To visually find the brain activity and ascertain the percentage values for ERD and ERS, the area of blue line with a square under red line with a rhombus represents ERD (power decrease), and the remainder area of green stands for ERS (power increase) during left and right hand MI (the time period 2 s after the cue appearance). The EEG reveals a significant ERD (Blue area) and relatively week ERS (Green area) over the contralateral side (such as C3 or C4). The feature reflects the change of signal characteristics, then it is expected to identify left or right hand MI.

In EMG mode, the common features of surface EMG such as mean absolute value, waveform length, zero crossing, slope sign change and mean absolute value slope are used as an input to a classification model for hand gestures recognition (Khokhar et al., 2010). Then there is a customized correspondence between 5 basic hand gestures and hand tasks according to what extent such a principle to improve the action recognition efficiency and accuracy rate, meanwhile hand gesture that are more easily performed matches well with hand action which is prioritized by frequency of use and importance, and the optimum relationship between hand gestures and actions is matched. If a subject performs the “Fist” hand gesture, this is recorded as a “Ball pinch” hand action, and the “Wave in,” “Wave out,” “Fingers spread,” and “Double tap” hand gestures correspond to “Tip pinch,” “Multiple tip pinch,” “Finger loosen up,” and “Cylindroid grip” hand actions, respectively (Boyali and Hashimoto, 2016). If the “Rest” hand gesture is performed, the mHMI is considered as “idle.”

### Performance Analysis of mHMI

**Figure 7:**
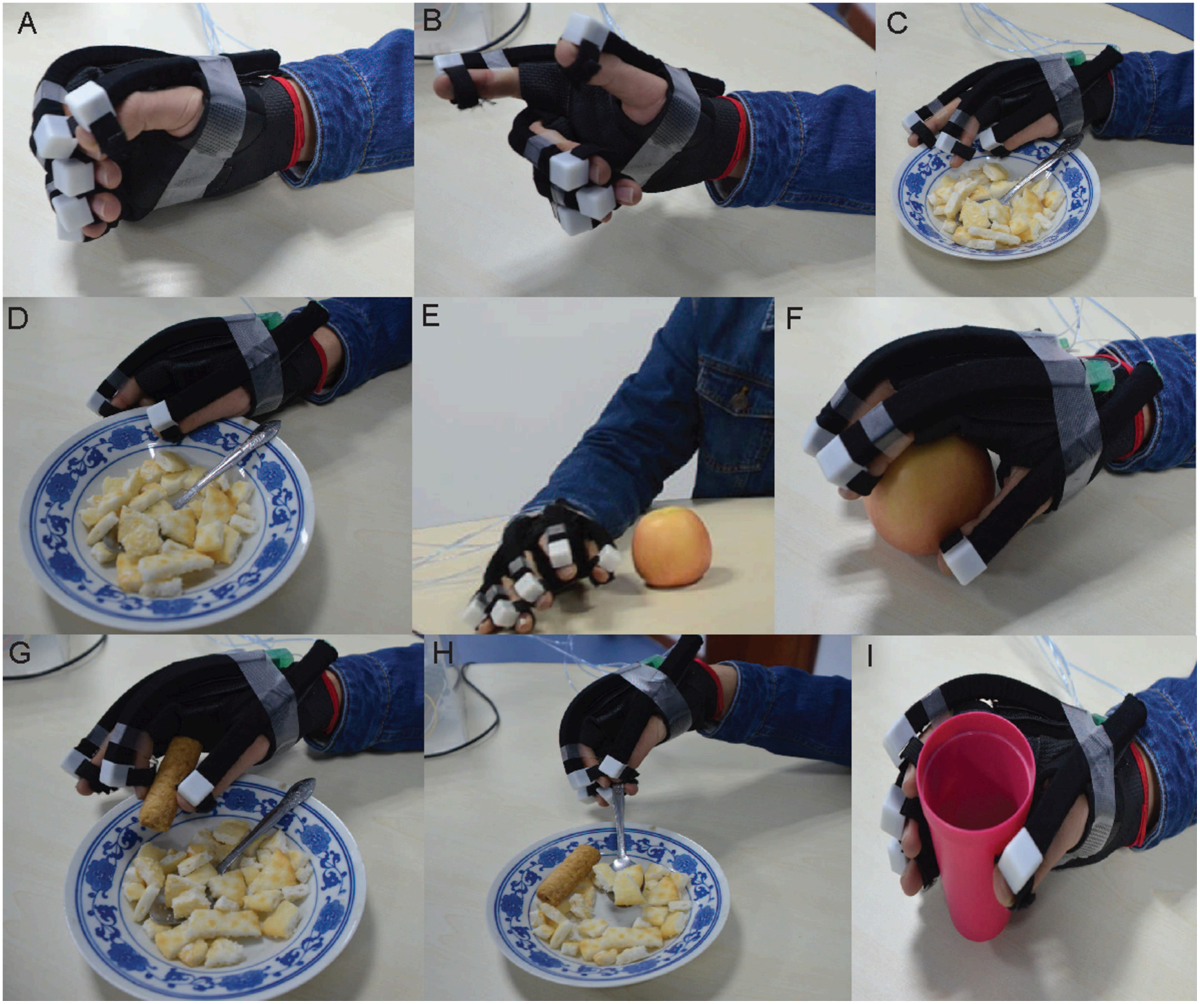
The hand action results are separately presented. A subject is capable of grasping various objects of their everyday live quickly according to his/her intention with assistance from soft robot. **(A,B)** Display separately the hand actions of fist and three finger grip in EEG mode. **(C,D)** Separately show the hand actions of push with bend five fingers, pull with pinch five fingers in EOG mode. **(E–I)** Illustrate hand actions of finger loosen up, ball pinch, tip pinch, multiple-tip pinch, and cylindroids grip, respectively in EMG mode.

Subject actively takes participate in the interactive home training to complete the hand movements based on the mHMI. Figures 7A–I separately illustrates different hand actions including fist, three finger grip, push with bend five fingers, pull with pinch five fingers, finger loosen up, ball pinch, tip pinch, multiple-tip pinch and cylindroids grip with the help of a soft robot prototype in daily life. To evaluate the performance of mHMI, the time and the number of correct or wrong actions for each action are all computed from the corresponding videotape in each run. The results of statistical analysis are introduced for each subject as shown in Table 2. From this, the performance of each subject can be analyzed on the basis of these results. The actions performed by the subjects take anywhere from 3.24 to 3.96 min, and with control speeds ranging from 15.08 to 18.52 times/min. The number of errors varies from 4 to 8, with a mean of 6.17 ±1.47 times. The ACC is about 91.83% ~ 96.12%, with an average of 93.83% ± 0.02. Clearly, S1 performed well with regard to both control speed and ACC, since this subject had access to a lot of training over a long period and was familiar with the control process. S4 also achieved a remarkable performance, adopting a cautious approach to obtain a good result and spending 3.96 s to finish the whole process, thus giving a classification rate of 95.29% with this control strategy.

**Table 2:** Performance form the six subjects of mHMI obtained with real-time control the soft robot.

**Subject**	**second per action (s)**	**Speed (actions/min)**	**Error times**	**ACC (%)**	**ITR (bits/min)**
S1	3.24	18.52	4	**96.12%**	54.82
S2	3.54	16.95	6	93.23%	46.50
S3	3.60	16.67	7	93.45%	45.99
S4	3.96	15.08	5	95.29%	43.66
S5	3.48	17.24	7	93.06%	47.11
S6	3.42	17.54	8	91.83%	46.39
AVG	3.54	17	6.17	93.83%	47.41
SD	0.24	1.14	1.47	0.02	3.82

*Subject 1, subject 2, … subject 6 are short for S1, S2, … S6, respectively. AVG means the corresponding average values, SD presents standard deviation. The result in bold number is the highest results among six subjects*.

The mHMI not only increases the number of control commands, but also enhances the classification accuracy by combining the EOG, EEG, and EMG modes. Here, the performances of the separate EOG, EEG, and EMG modes and of the multi-modal approach are assessed through an analysis of the average time, control speed, ACC, and ITR for all subjects in 50 runs. The performance is shown for each mode in Table 3. As can be seen, the average time is defined as the mean time of six subjects who finish the entire process from the start of one intention task to output the control command for each run in each mode. The number of actions is taken as the number of actions in a pre-defined manner for each run, with the EOG, EEG, EMG, and multi-modal modes having 2, 2, 6, and 10 action tasks, respectively. The control speeds of the EOG, EMG, multi-modal and EEG modes successively decrease, being 50, 42.86, 16.95, and 5.88 actions/min, respectively. And their respective ACC values separately are 94.23, 96.38, 91.46, and 93.83%. This also shows that the EMG mode gives greater accuracy than the EOG and EEG modes.

**Table 3:** The performance results of three single modes and multimodal in parameters of time, commands, speed, ACC and IRT.

**Parameters**	**EOG mode**	**EEG mode**	**EMG mode**	**Multimodal**
Time per action (s)	1.2	10.2	1.4	3.54
commands	2	2	6	10
Speed (actions/min)	50	5.88	42.86	16.95
ACC (%)	94.23%	91.46%	96.38%	93.83%
ITR (bits/min)	33.84	3.34	97.48	47.23

By considering the ITR of each mode, it is possible to evaluate the overall classification performance of each mode across six subjects. As can be seen from the above table, all subjects successful finish the control task using the mHMI, with a control speed of 17 ± 1.14 actions per minute, giving an ITR of 47.41 bits/min. The average ITR is significantly higher the EOG and EEG modes. Although the mHMI is not achieved the highest ITR value, it shows significantly more commands than the other single mode.

**Figure 8:**
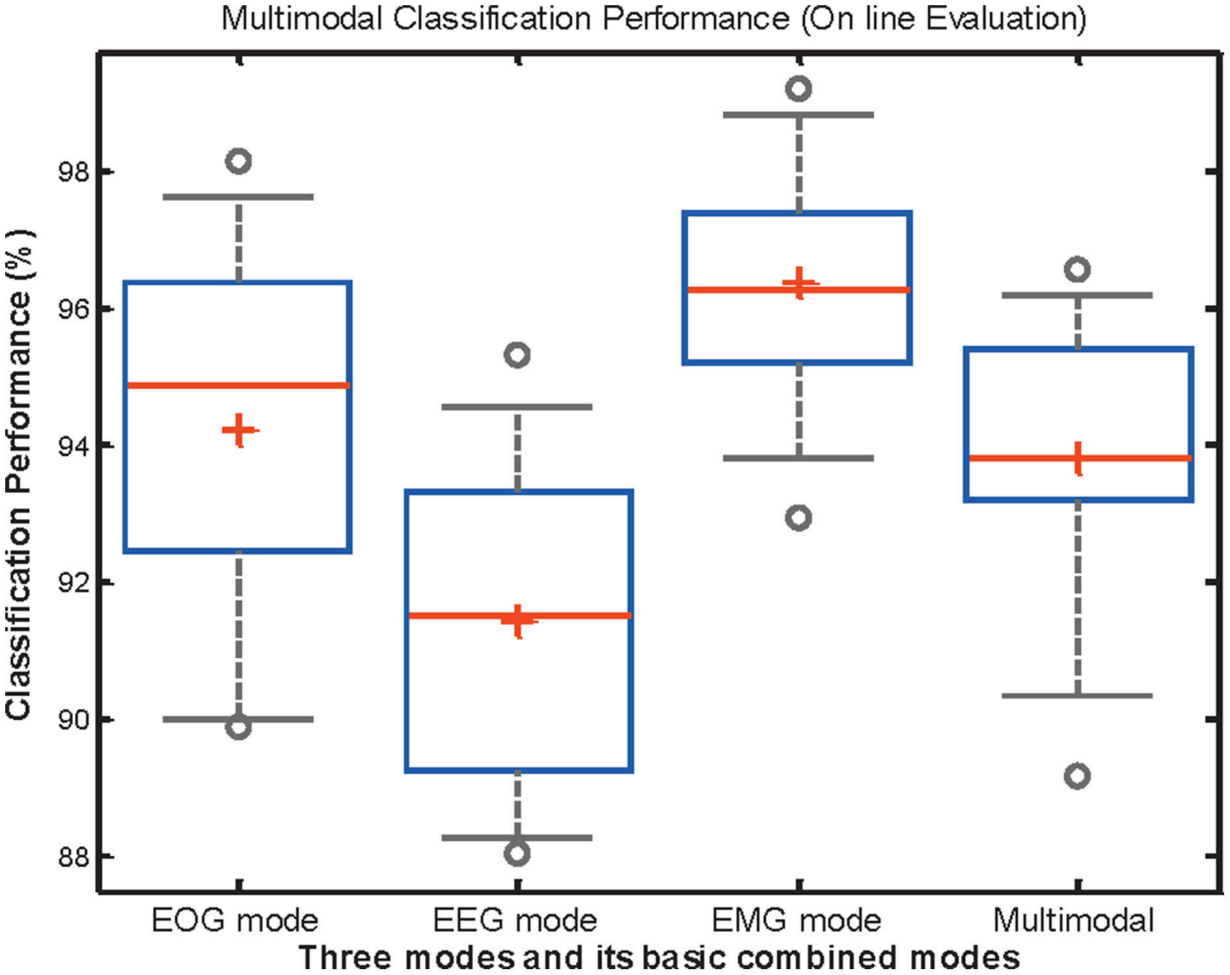
The distribution of classification performance in EOG, EEG, EMG, and theirs basic combined mode across all participants during on-line test. Box edges represent the interquartile range which is the distance between the 1st and 3rd quartiles, red line and cross is the median of interquartile range and the mean of minimum and maximum data values. Outlier points with circle stand for those that are greater than the interquartile range.

In order to analyze the differences between classification accuracy, the classification performance of EOG, EEG, EMG, and mHMI is presented in Figure 8. As can be observed, the ACC for each mode is above 88%, and even it reaches up to 98% in some cases. The EOG, EEG, EMG and multi-modal have their best performance and small deviation: 94.23% ± 0.0278, 91.46% ± 0.0229, 96.38% ± 0.0178, and 93.83% ± 0.0213, respectively. Those values possess a small standard deviation which implies that the classification performance is clustered close to its mean, and the corresponding system has stability and reliability. To further compare and analyze the significant differences between the modes, the *p*-value is assessed by a statistical study using one-way analysis of variance (ANOVA) test with IBM SPSS Statistics 19 (IBM Corporation, America) for classification accuracy. Regarding the ACC of each mode, there is a significant difference of each mode, there is a significant difference between the EMG and EEG modes [*F*_(1, 18)_ = 28.74, *p* = 0.000] and between EOG and EEG mode [*F*_(1, 18)_ = 5.87, *p* = 0.026], but no significant difference between the EOG and EMG modes [*F*_(1, 18)_ = 4.28, *p* = 0.053].

Specifically, the ACC between multi-modal and any single mode is compared by the ANOVA. There also exists considerable difference between multi-modal and EEG mode [*F*_(1, 18)_ = 5.73, *p* = 0.028], as well as EMG mode [*F*_(1, 18)_ = 8.43, *p* = 0.009]. Then it is not significantly influenced by EOG mode [*F*_(1, 18)_ = 0.13, *p* = 0.73]. However, the performance of the mHMI is represented by the mean value of all the evaluation parameters among different modal, it is not dominant in the various parameters when comparing with EOG, EEG, or EMG mode. Then it is significantly superior to the single modes with regard to control commands. In addition, it is further simplified to add commands and avoid the disadvantage of individual mode to the system, which is a further advantage of mHMI for a long period of real-time experiment.

### Comparison to Previous Work

Although the combination of EEG, EOG, and EMG modes in an mHMI is promising, there are very few BCI systems that have implemented this approach (Hong and Khan, 2017). However, in a similar approach, a system has been developed to control external devices or other services with different input modalities based on EEG, EOG, EMG, or motion signals. The combination of six different input signals gives an advantage in the number of input controls (total 11) in comparison with a single control/input mode, and the EEG-SSVEP method have an accuracy of 74%. All subjects are required to perform predefined tasks with each input method according to a sequential experimental paradigm (Edlinger et al., 2013). In contrast to our work, subjects are able to switch between modes to control desired actions at any time through double blinks, with a large number of control commands. A novel HMI to control a humanoid robot has also been presented and is the first interface that integrates GKP, EOG, and EMG to improve information capacity, the results shows that the mean accuracy is 86.7 ± 8.28% and the mean response time is 2.77 ± 0.72 s (Nam et al., 2014). This system detects four kinds of tongue movement or eye movements, as well as teeth clenching movements, and the subject controls the robot's performance of various actions by selecting from a predefined menu. In comparison with our work, the number of control commands is limited, and the inclusion of EEG in this system has not been investigated. In other work, a hybrid control approach at two levels has been used to control a 5 + 1 DOF robot from EEG, EOG, and EMG signals together with head movements via consumer-grade wearable devices, and the classification accuracy of hEOG, ERD/ERS and blink model is 73 ± 5% (Minati et al., 2016). A further approach to HMI has combined different modalities to potentially control 16 objects based on EEG, EOG, eye tracking, and EMG signals, and the prediction accuracy is 90.3 ± 8.8% (Novak et al., 2013). Although these two studies have integrated four or more different sensing technologies, they control a robot arm or objects based on combinations of four or more signals rather than integration of all signals, and the effect of the combination between three modes and a single mode have been investigated in terms of classification performance. However, no obvious increase is found in the number of control commands. Presently, there is less research on ITR of mHMI under the same conditions. Then there are some studies on ITR of mHMI as analogous to the current conditions as possible. The online result of hybrid HMI based on EMG and EOG is 43.3 bits/min (Buchwald and Jukiewicz, 2017), and the ITR of new hybrid EEG-EMG-BCI system is 18.43 bits/min in the process of writing some English letters with a robot arm (Gao et al., 2017). Our system translates three signals into 11 classes of control commands to control a soft robot with an accuracy of 93.83% which is outperformed the above mentioned system and its mean ITR is 47.41 bits/min, and it can be used to assist both healthy and disabled persons with high efficiency in classification performance compared with existing mHMI.

Overall, this comparison demonstrates clearly that our novel mHMI shows little similarity with other recent hybrid BCI systems with the capacity to control external devices for rehabilitation from a combination of three or more signals. To the best of our knowledge, the combination of EEG, EOG, and EMG signals has not been employed in previous studies. Furthermore, our prototype system has increased the number of control commands to a certain extent, and explored subject acceptance of an affordable wearable soft robot. Hence, it is more suitable for application by disabled persons using multiple rehabilitation devices for activities of daily living.

### Limitations

The evaluation of the mHMI has shown that the combination of three modes is feasible and effective in increasing the number of control commands. However, this prototype system still has several limitations.

First, the EEG mode impinges on the control speed of the mHMI in a synchronous manner. Subjects start to imagine left or right hand movement when they hear a “Thinking” sound from the computer, but it takes time to await the cue in this process, which affects the control speed of the system. Second, if the working pressure of the soft robot changes over time to fall below a barometric threshold, the stiffness change space is limited, although only to a certain extent (Yufei et al., 2017). Indirectly, this reduces the attainable strength of grasping actions, with further effects on applications such as lifting weights (Deimel and Brock, 2015). Third, because the subjects of the study were healthy individuals, rather than patients suffering from strokes or brain injuries, their movement imaginings may differ from those of patients with impaired motor functions (Ang et al., 2011). Fortunately, clinical trials of BCI rehabilitation therapy can detect the MI of patients to facilitate motor functional recovery (Ang and Guan, 2017). Similarly, eye movements of patients can be measured by EOG (Berger et al., 2006) and EMG have also been recorded from stroke patients (Cesqui et al., 2013). Thus, patients might be able to select certain modes of the mHMI to control the soft robot in a manner tailored to their individual conditions, and this would allow future investigations of the variation in classification performance between healthy and handicapped subjects. Finally, if a subject is not familiar with the operation of the mHMI, this affects the classification rate and control speed in the initial experiment. In addition, the experimental environment, including the subjects themselves, can have an impact on classification performance, for example if a subject is sweating or nervous, and the collection of EEG, EOG, and EMG data can be affected by different intensity of noise.

## Conclusion and Future Work

In this study, we have proposed a task-oriented approach to assistance and motor function training with the activities of daily living using the mHMI with robust real-time control of a soft robot through MI, eye movements, and hand gestures. The system integrates EEG, EOG, and EMG modes to increase the number of possible control commands to soft robot in his/her customary expressive way. Subjects select different modes with double blinks and execute various hand actions to indicate the required command easily, robustness and intuitively. The mHMI can detect 11 kinds of movement intention with an accuracy of 93.83% and an average ITR of 47.41 bits/min. The proposed mHMI real-time controls soft robot in friendly and convenient way to provide assistance to healthy or disabled persons with performing hand movement.

Future work will focus on the development of a portable, cheaper, fully asynchronous EEG/EOG/EMG-based mHMI and a synchronous multi-information acquisition system to improve control commands, control speed, ACC, and practicability. Meanwhile, the mHMI should be performed to assist chronic stroke patients in recovering their hand motor functions.

## Author Contributions

JZ conceived and designed the experiments. BW performed the experiments, analyzed the data, and was responsible for writing and revising the manuscript. CZ implemented the on-line control platform based on Visual C++ in Visual Studio and helped perform the experiments. YX analyzed and discussed the results. MW contributed to the important review of the study.

### Conflict of Interest Statement

The authors declare that the research was conducted in the absence of any commercial or financial relationships that could be construed as a potential conflict of interest.
